# NODDI and Tensor-Based Microstructural Indices as Predictors of Functional Connectivity

**DOI:** 10.1371/journal.pone.0153404

**Published:** 2016-04-14

**Authors:** Fani Deligianni, David W. Carmichael, Gary H. Zhang, Chris A. Clark, Jonathan D. Clayden

**Affiliations:** 1 Developmental Imaging and Biophysics Section, Institute of Child Health, University College London, London, United Kingdom; 2 Centre for Medical Image Computing, Department of Computer Science, University College London, London, United Kingdom; Universiteit Gent, BELGIUM

## Abstract

In Diffusion Weighted MR Imaging (DWI), the signal is affected by the biophysical properties of neuronal cells and their relative placement, as well as extra-cellular tissue compartments. Typically, microstructural indices, such as fractional anisotropy (FA) and mean diffusivity (MD), are based on a tensor model that cannot disentangle the influence of these parameters. Recently, Neurite Orientation Dispersion and Density Imaging (NODDI) has exploited multi-shell acquisition protocols to model the diffusion signal as the contribution of three tissue compartments. NODDI microstructural indices, such as intra-cellular volume fraction (ICVF) and orientation dispersion index (ODI) are directly related to neuronal density and orientation dispersion, respectively. One way of examining the neurophysiological role of these microstructural indices across neuronal fibres is to look into how they relate to brain function. Here we exploit a statistical framework based on sparse Canonical Correlation Analysis (sCCA) and randomised Lasso to identify structural connections that are highly correlated with resting-state functional connectivity measured with simultaneous EEG-fMRI. Our results reveal distinct structural fingerprints for each microstructural index that also reflect their inter-relationships.

## Introduction

Microstructural indices obtained from Diffusion Weighted Images (DWI) are hard to interpret because of the difficulties in disentangling the contribution of different tissue compartments [1, 2]. For example, tensor-based scalar invariants, such as fractional anisotropy (FA) and mean diffusivity (MD), reflect several biophysical properties of neuronal cells, such as neuronal density, fibre orientation dispersion, axonal diameter and degree of myelination [3, 4]. Neurophysiological interpretation of these parameters is further complicated because of variability in neuronal packing and dispersion across white matter fibres.

Recently, more biophysically plausible models have been developed to allow a better description of the underlying tissue microstructure. Neurite Orientation Dispersion and Density Imaging (NODDI) exploits multi-shell DW-MRI acquisition protocols to model the diffusion signal as the contribution of three compartments: intracellular, extracellular and cerebrospinal fluid [5]. NODDI is an attractive biophysical model for describing the diffusion process because it can be easily adopted and implemented on clinical systems [5–7].

One way of examining the neurophysiological role of these microstructural indices is to look into how they relate to brain function. In fact, relating functional and structural connectomes has become an increasingly attractive way to explore commonalities between multimodal measurements [8–14]. The success of these approaches is underlined by the fact that there is strong coupling between functional and structural connectivity [11, 15, 16]. Furthermore, these commonalities are driven by neurophysiological factors, and are unlikely to result from correlated artefacts or noise between the two modalities.

In our previous work, we have used simultaneous recordings of fMRI and EEG to relate resting-state, whole-brain connectomes across frequency bands [17]. fMRI data represent blood oxygen level-dependent (BOLD) contrast, which is an indirect measure of brain function and limits the temporal resolution of neuronal fluctuations observed. On the other hand, EEG measures electrical activity, which results directly from brain function. However, localising the signal is challenging and has limited spatial accuracy. Therefore, combined recordings of EEG and fMRI signals characterise the underlying neurophysiological events more accurately than either of these modalities separately. We investigated neuronal activity in different frequency bands because they subserve different roles. For example occipital alpha (*α*) and beta (*β*) bands have been related to resting-state networks observed with fMRI [18, 19]. Filtering of the EEG signal into different frequency bands also increase the signal-to-noise ratio and improves source localisation [20].

We have previously derived fMRI connectomes based on the precision matrix of the rs-fMRI time-series. EEG functional connectomes were derived for each of five frequency bands (*δ* (1–4Hz), *θ* (4–8Hz), *α* (8–13Hz), *β* (13–30Hz) and *γ* (30–70Hz)), based on the inverse covariance matrix of the band-limited power of the source-localised EEG time series, averaged within brain regions [17]. In that work, the geodesic distance between fMRI and EEG brain connectomes was smaller for the *θ* band. Furthermore, we had exploited a statistical framework based on sparse Canonical Correlation Analysis (sCCA) to model the relationship between multi-modal measurements of functional connectivity [21–23]. This allowed us to assign a probability for each connection in each modality that reflected how significant it was in mapping one modality to the other via sCCA. This process is also called model identification in machine learning, and it is used to select the model parameters, fMRI-EEG links, that we can reliably consider to be nonzero [14].

Here, we have the opportunity to explore the relationship between functional brain connectomes and structural brain connectomes based on the weighted average of microstructural indices along fibre pathways, extracted using tractography. FA and MD are derived from the traditional diffusion tensor model. On the other hand, we use NODDI to derive: *i)* intra-cellular volume fraction (ICVF), which is a marker of neuronal density, *ii)* orientation dispersion index (ODI), which characterises the angular variation of neurites, *iii)* the concentration parameter (*κ*) that measures the extent of orientation dispersion, and is analytically related to ODI in a nonlinear way, and *iv)* the volume fraction of the isotropic compartment (ISO). We are interested in modelling the relationship between functional and structural connectomes to understand how different microstructure indices are related to brain function. To this end we identify the structural connections that are strongly related to functional connectivity, with selection rate significantly above chance.

Our model identification results show that in ICVF and MD the structural connections that mostly underpin functional connectivity are intra-hemispheric connections, whereas in ODI, *κ* and WFA, structural connections that underpin functional connectivity are mostly interhemispheric. The structural connections with the highest selection rate in predicting functional connectomes derived from fMRI are also selected with high probability in predicting EEG connectomes. Further pair-wise correlation analysis of the microstructural indices across white matter connections demonstrates that FA correlates with ODI (*R*^2^ = 0.642), *κ* (*R*^2^ = 0.436), WICVF (*R*^2^ = 0.219) and MD (*R*^2^ = 0.122). ODI and *κ* are correlated (*R*^2^ = 0.588), whereas MD also correlates with ICVF (*R*^2^ = 0.49). A relationship also exists between ICVF and ISO (*R*^2^ = 0.131). These relationships between microstructural indices support the model identification results obtained.

## Methods

### Imaging

Imaging data from 17 adult volunteers (11 males, 6 females, mean age: 32.84 ± 8.13 years) were acquired in a Siemens Avanto 1.5 T clinical scanner using a self-shielded gradient set with maximum gradient amplitude of 40 mT m^−1^. Data were acquired in two sessions:

*i)* Simultaneous resting-state EEG-fMRI were acquired with a standard 12 channel coil. The subjects had their eyes open and were asked to remain awake and fixate on a white cross presented on a black background. Subjects were asked to remain still and their head was immobilised using a vacuum cushion during scanning. The fMRI imaging acquisition was based on a T2*-weighted gradient-echo EPI sequence with 300 volumes, TR/TE = 2160/30 msec, 30 slices with thickness 3.0 mm (1mm gap), effective voxel size 3.3 × 3.3 × 4.0 mm, flip angle 75°, FOV 210×210×120 mm. Scalp EEG was recorded during the MRI scanning using a 64 channel MR-compatible electrode cap (BrainCap MR, Gilching, Germany) at native frequency of 1000 Hz. The electrodes were arranged according to the modified combinatorial nomenclature, referenced to the FCz electrode. The electrocardiogram (ECG) was recorded, and EEG and MR scanner clocks were synchronised. A T1-weighted structural image was also obtained.

*ii)* Structural data were acquired with a 32-channel head coil. The NODDI sequence optimisation on the 1.5 T scanner follows the experiment design procedure in Alexander et al. [24] with the NODDI model [5]. The a priori model parameter settings are: intracellular volume fraction *f* = 0.3, 0.5, 0.7, intrinsic diffusivity *d* = 1.7 × 10^−9^ *m*^2^ *s*^−1^, perpendicular diffusivity set according to the tortuosity constraint [25], Watson concentration parameter *κ* = 0.5, 2, 8, 32. The scanner-specific sequence settings are: *T*2 = 80 *m* *s*, *G*_*max*_ = 40 *m* *T*/*m*. Finally, we target a total of 109 measurements to have a total acquisition time of around 16 minutes. The optimisation divides the measurements into three HARDI shells with *b* = 2400 s mm^−2^ (60 noncollinear gradient directions and six *b* = 0 images), *b* = 800 s mm^−2^ (30 noncollinear gradient directions and three *b* = 0 images) and *b* = 300 s mm^−2^ (9 noncollinear gradient directions and one *b* = 0 image) were acquired with a voxel matrix of 96×96, 60 contiguous axial slices, each 2.5 mm thick, with 240×240×150 mm field of view (FOV), voxel size of 2.5×2.5×2.5 mm and TR/TE = 8300/98 ms. We have kept the same TE across shells to avoid differences in T2 effects that would need to be accounted for the diffusion model, and thus we avoided adding complexity.

High resolution T1-weighted whole-brain structural images were also obtained in both sessions with voxel size of 1.0×1.0×1.0 mm, TR/TE = 11/4.94 ms, flip angle 15°, FOV 256×256×256 mm, voxel matrix 176 × 216 and 256 contiguous slices. The mean interscan interval was 35 ± 41.6 days.

Ethical approval was obtained from the UCL Research Ethics Committee (project ID:4290/001) and informed written consent was obtained from all subjects.

### Constructing Functional Connectomes

fMRI and EEG connectomes were derived according to our previous work [17]. Briefly, the two T1-weighted images obtained per subject were processed with FreeSurfer to obtain a grey matter (GM) parcellation into 68 cortical regions [26]. The first five volumes of the rs-fMRI were removed to avoid T1 effects and images were preprocessed with FSL [27]. fMRI time-series were averaged within each GM region and the precision matrix was used to estimate fMRI functional connectomes [28]. EEG was corrected offline for scanner [29] and cardiac pulse related artefacts [30] using Brain Vision Analyzer 2 (Brain Products, Gilching, Germany). Subsequently, it was down-sampled to 250 Hz and further analysis was carried out with SPM12b (www.fil.ion.ucl.ak.uk) [31]. This analysis included: *i)* band pass filtering into five bands: *δ* (1–4Hz), *θ* (4–8Hz), *α* (8–13Hz), *β* (13–30Hz) and *γ* (30–70Hz). *ii)* Segmentation into (fMRI) TR epochs (2.16 sec). *iii)* Definition of a head model based on a standard template head model in SPM. *iv)* Definition of forward model based on the three-shell boundary element model. *v)* Source localisation based on beamforming SPM toolbox [32, 33]. For each GM cortical region, the EEG signal is projected from sensor space to points randomly drawn from the region, independently for each subject. *vi)* Estimation of Hilbert envelope across the whole source-localised time series. *vii)* Averaging the Hilbert envelopes within each GM region and estimating the precision matrix to represent EEG functional connectomes.

Functional connectomes across all subjects, derived from both fMRI and EEG data, can be found in the supplementary material, S1 Data. These are provided as the corresponding precision matrices.

### Constructing Structural Connectomes

We used TractoR for preprocessing of the diffusion weighted images [34]. This involves converting DICOM files into a 4D NIfTI file, identifying the volume with no diffusion weighting to use as an anatomical reference, creating a mask to identify voxels which are within the brain, and correcting the data set for eddy current induced distortions. The last two stages are performed using FSL. Furthermore, the gradient vectors are corrected retrospectively to account for eddy current induced distortions [34].

Fractional anisotropy (FA) and mean diffusivity (MD) were estimated with TractoR based on a tensor model fitted in each voxel. For the NODDI data we concatenated the three shells with *b*-values of 2400 s mm^−2^, 800 s mm^−2^ and 300 s mm^−2^ in one shell, along with the *b* = 0 images acquired in each shell. Subsequently, we used TractoR, which wraps FSL, for eddy current correction along the concatenated volume of images. We used the NODDI Matlab toolbox to extract ICVF, ODI, ISO and *κ*[5]. A ball and two sticks multi-compartment fibre model was fitted to the shell of diffusion data acquired with *b* = 2400 s mm^−2^, using the Bayesian Estimation of Diffusion Parameters Obtained using Sampling Techniques (BEDPOSTX) algorithm in FSL [35]. Subsequently, we ran probabilistic tractography, implemented in TractoR, for each dataset. We seeded 100 streamlines from each white matter voxel, and tracking was terminated when both ends of a track reached a cortical target region.

Structural brain connectomes are described as graphs, with nodes corresponding to ROIs and edges defined based on either the number of streamlines or as weighted averages of microstructural indices. In weighted averages, weights reflect the number of streamlines that pass through each voxel of the tract. Weighted averages minimise the influence of voxels that are unlikely to belong to the tract. Below we describe the construction of each microstructural index: *i)* As the number of fibres that connect the regions, divided by the average number of voxels within the two end-point ROIs (NSTREAMS). *ii)* As the weighted average of FA along the streamlines that connect the two regions (WFA). *iii)* As the weighted average of MD along the streamlines that connect the two regions (WMD). *iv)* As the weighted average of ICVF along the streamlines that connect the two regions (WICVF). *v)* As the weighted average of ODI along the streamlines that connect the two regions (WODI). *vi)* As the weighted average of ISO along the streamlines that connect the two regions (WISO). *vii)* As the weighted average of the *κ* along the streamlines that connect the two regions (Wkappa). We used an anisotropy threshold of *FA* > 0.2 for seeding in tractography to reduce spurious streamlines. In this way, we produce seven structural brain connectomes for each subject.

Structural connectomes across all subjects and microstructural indices can be found in the supplementary material, S1 Data.

### Predictive modelling of functional from structural connectomes based on sCCA

Building upon our previous work, we are interested in sparse sets of associated variables that maximise the relationship between functional **Y** and structural connectomes **X** across subjects [17]. **Y** is an *k*_*y*_ × *m* matrix, with *k*_*y*_ the number of functional connection across all subjects and *m* the number of subjects. **X** is an *k*_*x*_ × *m* matrix with *k*_*x*_ the number of structural connections across subjects. Regularisation via the introduction of sparsity constraints is important in situations where the number of observations is much lower than the number of variables [14, 21, 36]. Therefore, we adopt sparse CCA (sCCA), which aims to find two sparse canonical vectors, *u* and *v*, subject to *L*_1_ penalties, such that the projections of **X** and **Y** onto these vectors, respectively, are maximally linearly correlated [21, 22]. More details about this framework can be found in our previous work [17].

#### Identification of the most relevant connections based on sCCA

Identification of the most relevant connections is important to provide biological interpretation of the mapping of structural to functional connections. However, devising a statistically sound way to accept or reject the null hypothesis is challenging because of the complexity of the underlying inference problem [14, 17]. To this end, in [17] we modified sCCA based on the randomised Lasso principle [23].

Here, we adopt this approach and we use bootstrap with resampling to extract the connections that are consistently selected. In each bootstrap iteration we set the number of canonical variates to *K* = 1, which simplifies interpretation. Therefore, we estimate the probability of selecting a connection as the number of times the connection has been selected over the number of total bootstrap iterations. Note that the parameters that control sparsity remain the same through out the bootstrap iterations and across all microstructural indices. In other words, the sparsity of the sCCA loadings remains constant, and reflects the probability of a connection by chance. Subsequently, we use the lower and upper tail of the binomial distribution to test whether a connection is selected or rejected significantly above chance, respectively.

## Results

**Fig 1:**
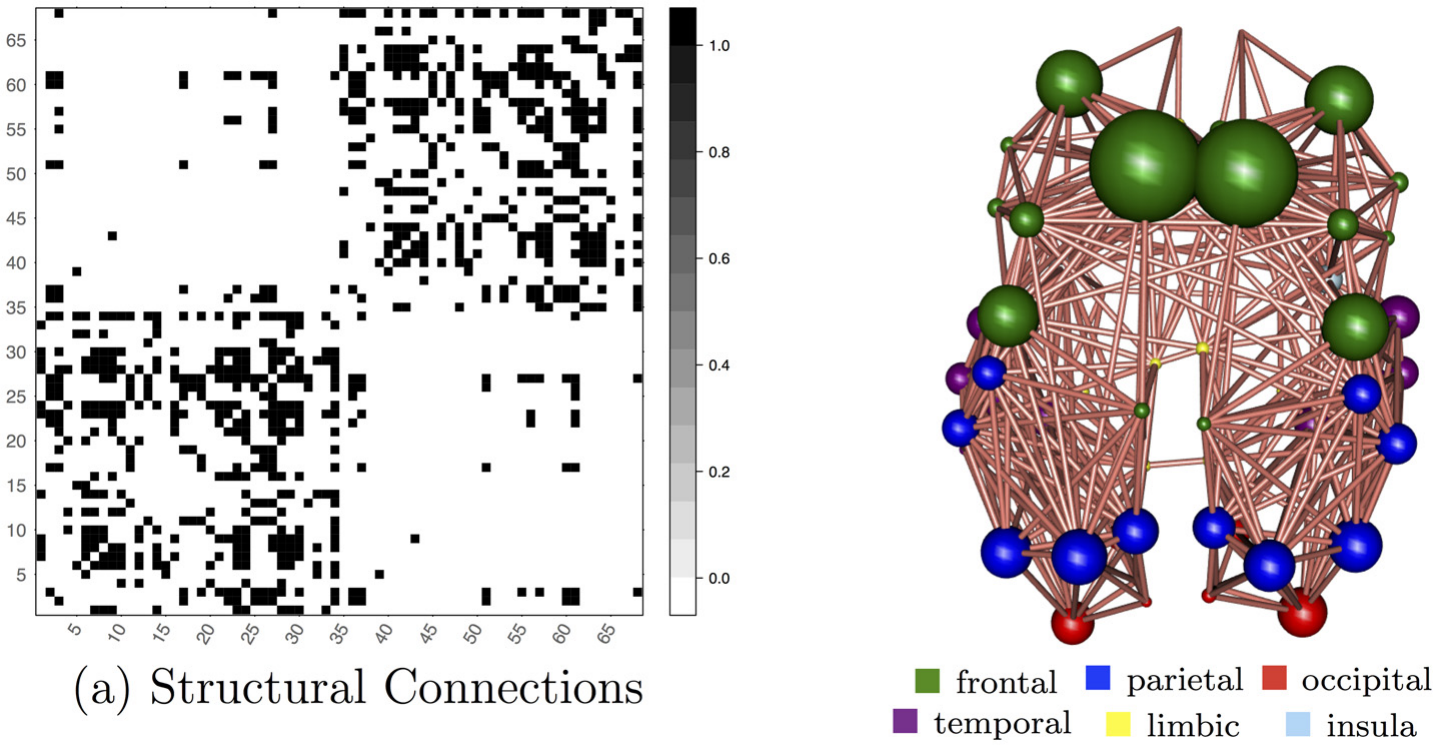
Structural connections that are present across all subjects and used in the sCCA framework. The subfigure on the left shows the structural connections represented as a symmetric, 68 × 68 binarised connectivity matrix. The bottom left matrix quadrant represents connections within the left hemisphere, the top right represents connections within the right hemisphere, and the remaining quadrants represent interhemispheric connections. The subfigure on the right shows the same connections represented as a three-dimensional graph. Each region is represented as a blob with size proportional to the corresponding size of the FreeSurfer region.

Fig 1 shows the structural connections that are present across all subjects and used in the sCCA framework. Note that sCCA standardises the connectivity values across each connection, so that they have zero mean and unit standard deviation. It is not appropriate to represent missing connections with zeros, since microstructural indices cannot be calculated in these cases. Instead connections that are missing in any subject are excluded. The remaining connections are shown with both a symmetric, 68 × 68 binarised connectivity matrix and a three-dimensional graph. In three-dimensional graphs, each region is represented as a blob with size proportional to the corresponding size of the FreeSurfer region. The bottom left matrix quadrant represents connections within the left hemisphere, the top right represents connections within the right hemisphere, and the remaining quadrants represent interhemispheric connections.

**Fig 2:**
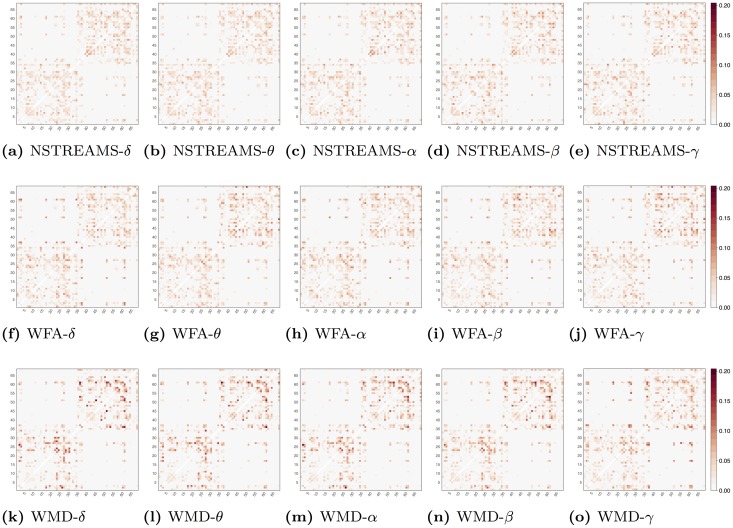
Probability maps for tensor-based microstructural indices, respectively. These probability maps are represented as 68 × 68 square arrays with a value in each cell that represents the probability of each structural connection to be selected during randomised Lasso bootstrap iterations. NSTREAMS represent that the underlying structural connectivity matrices were derived based on the number of streamlines divided by the average number of voxels within the two end-point ROIs. WFA and WMD reflect that the underlying structural connectivity matrices are derived as a weighted average of the fractional anisotropy (FA) and the mean diffusivity (MD) along the streamlines, respectively. On the other hand, *δ*, *θ*, *α*, *β* and *γ* represent that the underlying EEG functional connectivity matrices have been derived based on bandpass filtering in five frequency bands 1–4Hz, 4–8Hz, 8–13Hz, 13–30Hz and 30–70Hz, respectively.

**Fig 3:**
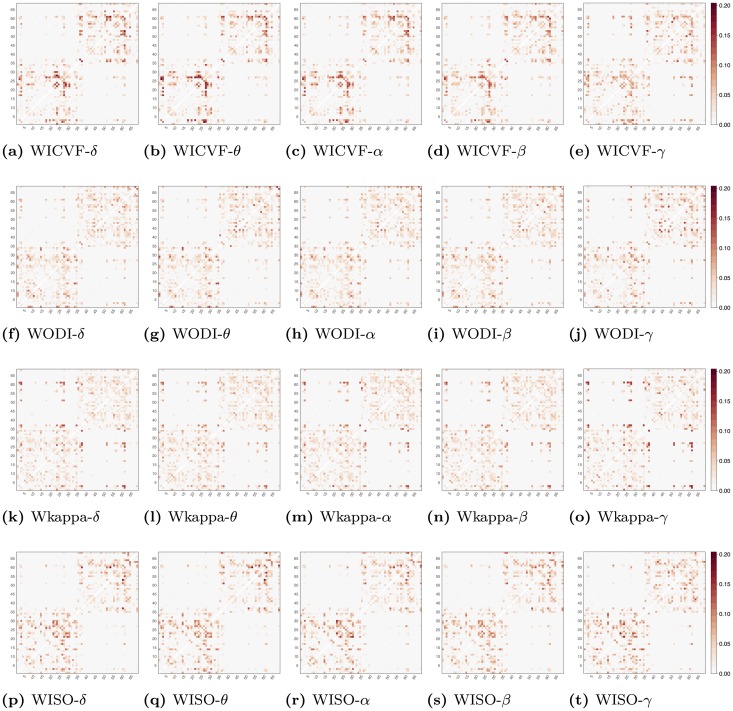
Probability maps for NODDI-based microstructural indices, respectively. These probability maps are represented as 68 × 68 square arrays with a value in each cell that represents the probability of each structural connection to be selected during randomised Lasso bootstrap iterations. WICVF, WODI, WISO and Wkappa reflect that the underlying structural connectivity matrices are derived as a weighted average of the intra-cellular volume fraction (ICVF), the orientation distribution index (ODI), the isotropic compartment (ISO) and the kappa parameter, respectively, along the streamlines. On the other hand, *δ*, *θ*, *α*, *β* and *γ* represent that the underlying EEG functional connectivity matrices have been derived based on bandpass filtering in five frequency bands 1–4Hz, 4–8Hz, 8–13Hz, 13–30Hz and 30–70Hz, respectively.

Figs 2 and 3 show probability maps for tensor and NODDI microstructural indices, respectively. These probability maps are represented as 68 × 68 symmetric, square arrays with a value in each cell that represents the probability of each structural connection to be selected. The probability values have been estimated as the ratio of the times each connection has been selected over 1000 bootstrap with replacement in a sCCA framework with randomised Lasso.

From an average of 453 ± 16 structural connections across bootstrap iterations 20 ± 1.8 are selected in each iteration. Based on a uniform distribution each connection has a probability of 0.044 to be selected by chance. For each of the probability maps presented in Figs 2 and 3, we use the upper bound of the lower tail of a binomial distribution to reject connections with probability significantly lower than chance (*p* < 0.05). Furthermore, we use the lower bound of the upper tail of the binomial distribution to highlight connections with probability of selection significantly above chance (*p* < 0.05).

**Fig 4:**
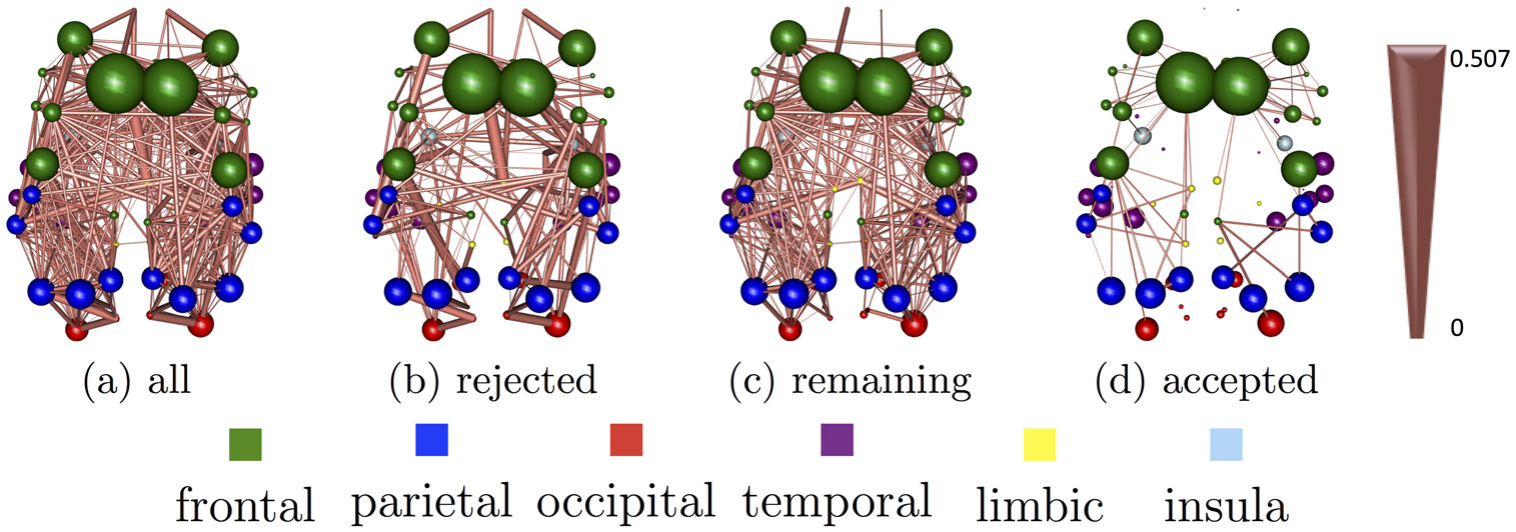
An example of the application of the binomial distribution on the structural WICVF-weighted connections derived with sCCA between WICVF and the *δ* EEG connectomes. We show the averaged canonical correlation coefficient weights of the structural connections. Fig 4a shows all the available structural connection, Fig 4b shows the connections that are rejected with probability significant above chance, Fig 4c shows the remaining connections once we remove the rejected connections and Fig 4d shows the connections that are selected with probability significantly above chance.

Fig 4 shows a representative example of the application of the binomial distribution on the probabilities of structural connections derived with sCCA across all bootstrap iterations. In particular, we show structural WICVF-weighted connections derived with sCCA between WICVF and the *δ* EEG connectomes. We show the average canonical correlation coefficient weights of the structural connections. From left to right, we see all the connections, Fig 4a, the connections that are rejected with probability significant above chance, Fig 4b, the remaining connections once we remove the rejected connections, Fig 4c and the connection that are selected with probability significantly above chance, Fig 4d.

**Fig 5:**
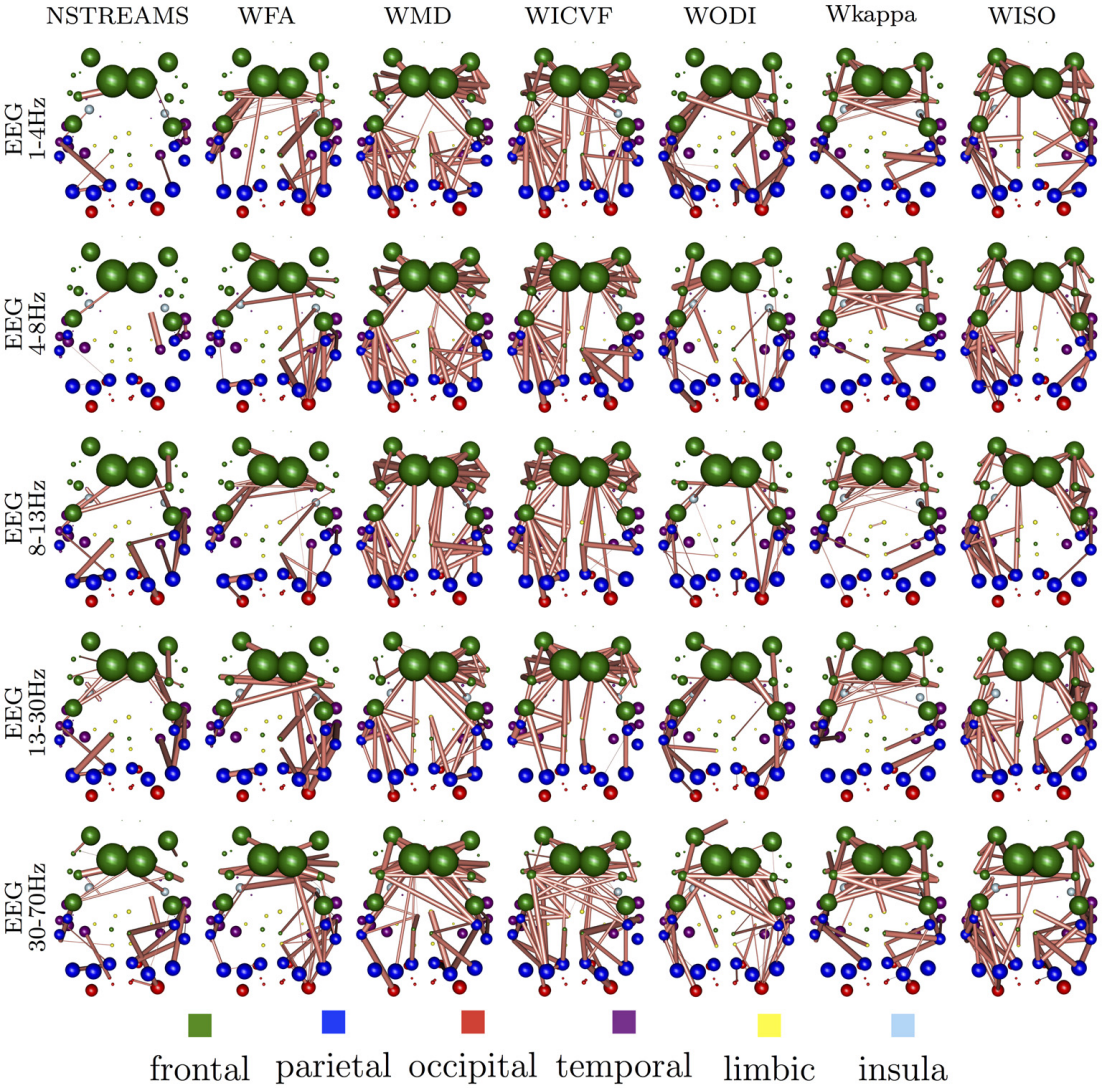
Structural connections that have been selected with probability significant above chance for each pair of electrophysiological band and microstructural index.

**Fig 6:**
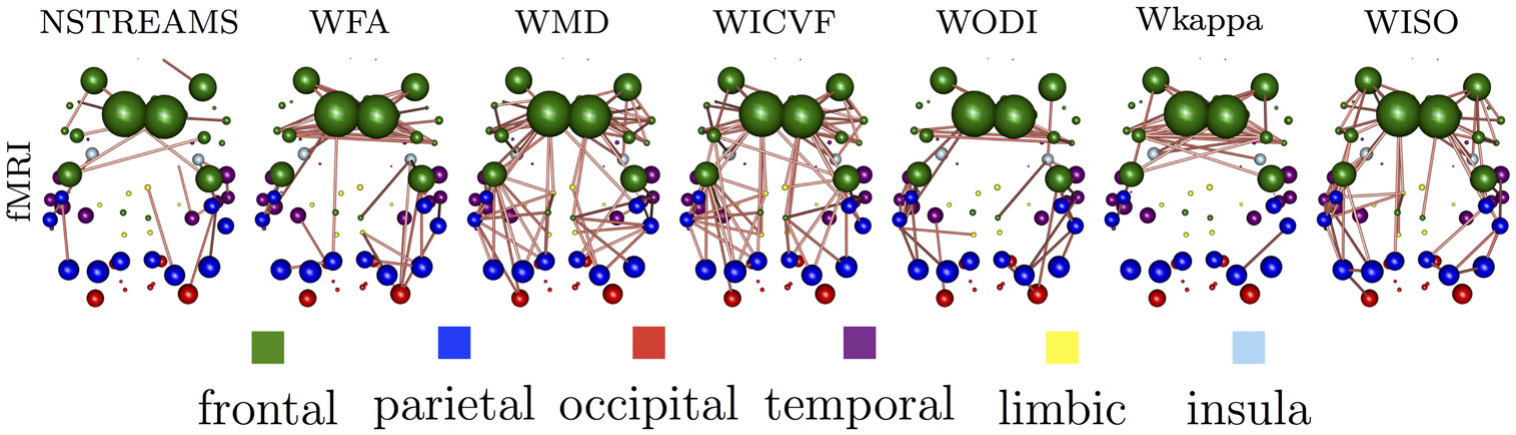
Structural connections that have been selected with probability significant above chance for rs-fMRI connectomes and each microstructural index.

We follow this process for each combination of electrophysiological band and microstructural index. Fig 5 summarises the structural connections that have been selected with probability significant above chance. We observe that in WMD and WICVF the relationship between function and structure is mediated by intra-hemispheric connections, whereas in WFA, WODI and Wkappa interhemispheric connections play a significant role. Similar patterns of selected structural connections emerge when we examine the pair-wise relationships of microstructural indices and rs-fRMI connectomes, Fig 6.

**Fig 7:**
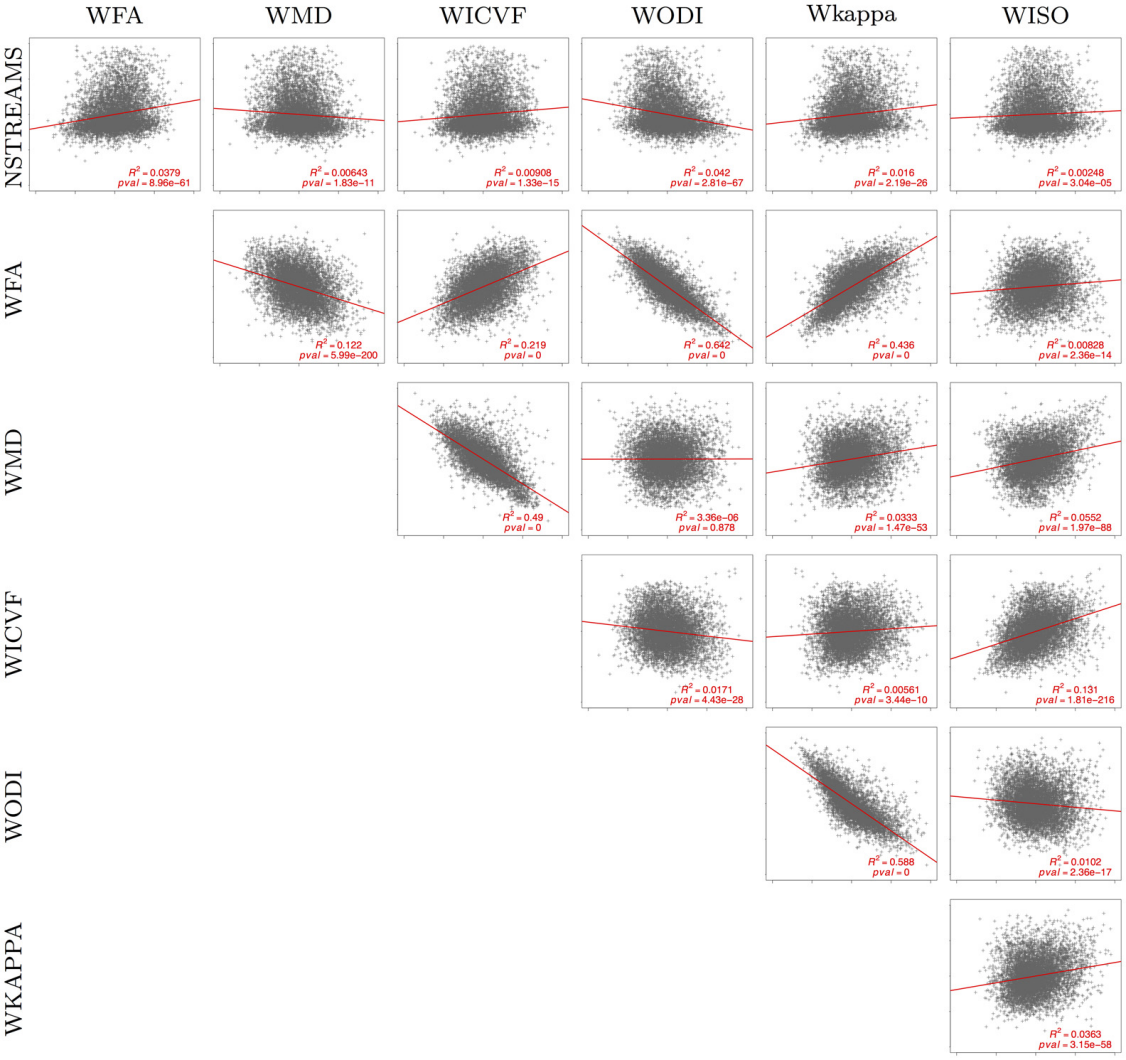
Pairwise relationships of microstructural indices.

To better understand how microstructural indices relate across subjects, we concatenated the connectivity values across all subjects into columnwise vectors for each index. Fig 7 shows a scatter plot of each pair of microstructural indices. We observe that WFA is relatively correlated with Wkappa and anti-correlated with WODI, whereas WMD is anti-correlated with WICVF. In particular, pair-wise correlation analysis of the averaged microstructural indices across white matter connections demonstrates that FA correlates with ODI (*R*^2^ = 0.642), *κ* (*R*^2^ = 0.436), WICVF (*R*^2^ = 0.219) and MD (*R*^2^ = 0.122). ODI and *κ* are correlated (*R*^2^ = 0.588), whereas MD also correlates with ICVF (*R*^2^ = 0.49). A relationship also exists between ICVF and ISO (*R*^2^ = 0.131).

## Discussion

We have developed a framework based on statistical prediction to relate functional and structural connectomes across microstructural indices. We used a multivariate approach based on sCCA that allows detection of linear relationships between two sets of variables and it is closely related to multivariate multiple regression analysis [37]. sCCA only encodes linear relationships between structure and function. An advantage of this is that it enables a considerable reduction in computational complexity. Recent work also supports the idea that linearisation allows a compact relationship between the covariance matrix of functional and structural connectivity and it results in statistically meaningful conclusions [11, 14]. Note that a linear relationship has also been found between in vivo measurements of axon diameter from DW-MRI and conduction velocity from electrophysiological data [38].

Since only a few measurements/subjects are available to infer relationships for hundreds of connections, we chose sparse CCA for regularisation. Regularisation is introduced via sparsity constraints for both the prediction and the predicted variables. Here, variables are the structural and functional brain connections derived from the vectorisation of the upper triangular part of the corresponding connectivity matrices.

Subsequently, we used a randomised Lasso procedure to assign a probability for each connection, which reflects the fraction of times it has been selected during all bootstrap iterations. Randomised Lasso improves the sparse recovery properties of Lasso because it decreases the dependence of the selected coefficients on the initial choice of regularisation parameters. Finally, we devised a null hypothesis based on the binomial distribution, which allows us to both identify connections that are selected significantly above chance and to also reject connections with a probability significantly lower than chance. This is a contribution that improves upon our previous work as it introduces an objective way to control the false positive rate.

Our results show that there is a strong relationship between WODI and WFA as well as between WICVF and WMD. In fact, pairwise correlations of the microstructural indices reveal that FA is related to ODI and *κ*, whereas MD is related both to ICVF and ISO. These are expected results given what these parameters measure, which it is highlighted further below. Note also that ODI is related to *κ* via a nonlinear relationship: ODI = 2/*π* ⋅ arctan(1/*κ*).

It is significant that these relationships between microstructural indices also emerge in the spatial pattern of structural brain connections selected as the most predictive of functional connectivity. For example, the structural brain connections derived from either WICVF or WMD that contribute mostly to the prediction of functional connectomes are predominately intrahemispheric connections. On the other hand, the structural brain connections derived from either WODI, Wkappa and WFA that contribute the most to the prediction of functional connectomes are predominately interhemispheric connections. The fact that these relationships emerge from a data-driven analysis of the relationship between functional and structural connectomes for both fMRI and EEG brain connectomes provides substantial evidence for the face validity of the suggested approach.

Our analysis is important for several reasons. It highlights that FA and MD as well as ICVF, ODI and ISO provide complementary information, since the identified structural fingerprints are substantially different. Pair-wise correlations of microstructural indices offer limited information about brain connectivity. It is only indicative of averaged whole-brain relationships of structural connectivity. The proposed methodology not only relates structural with functional brain connectivity but it also provides information about which specific connections are involved in this relationship.

These results are consistent with literature that shows that interhemispheric FA correlates with the underlying resting-state functional connectivity in several types of pathological networks, such as in Alzheimer’s disease [39] and migraine patients [40]. In previous work, we have also found that interhemispheric connections were most influential in the principal component that predicted IQ across late childhood and adolescence, irrespective of age or gender [41]. Although, FA has been used extensively as a measure of white matter integrity, it is under debate what are the exact neurophysiological factors that contribute to the anisotropy observed [42]. For example, cell membranes, myelin sheath, axonal density and axonal diameter as well as structure of surrounding tissues all have an impact on anisotropy. Recent literature also supports the idea that FA is sensitive both to the cells anisotropic structure and their orientation dispersion [43].

On the other hand, ODI is designed to capture mainly the orientation dispersion of neurites. Some dependence of ODI on axon diameter might still exist, since NODDI has simplified the diffusion model by omitting the axon diameter of neurites in order to achieve a clinically feasible imaging protocol [5, 44]. Here we demonstrated that both neurite density and dispersion contribute to FA, whereas ODI resolves this dependency.

We observed profound similarity between MD and ICVF structural fingerprints related to resting-state function. MD changes have been related to neural plasticity, though the cellular underpinnings are also unknown. Several studies provide evidence of experience-dependent white matter changes that may not be driven by changes to myelination but probably reflect cellular adaptations, such as astrocyte swelling, synaptic changes, dendritic spine changes, and so on [45, 46].

Our results might indicate that depending on neuronal packing and orientation dispersion, different parameters of the same diffusion model become more or less relevant in characterising the link between function and structure. This might have important implications for studies that examine brain connectivity based on tractography and demonstrate differences in FA due to a disease process or ageing. For example, if FA characterises function better in interhemispheric connections then results might be biased towards finding differences in those connections. This does not preclude that differences in actual tissue microstructure do not exist in other connections. But it rather indicates that FA is not sensitive enough to capture them.

Furthermore, recent evidence suggests that the relationship between functional and structural connectomes changes during disease processes. For example, in Alzheimer disease a significant decrease in the coupling between functional and structural connectivity was observed [47]. In schizophrenia, this coupling was increased though there was significant reduction in both functional and structural connectivity [48]. These results may reflect disease-specific mechanisms but their interpretation is not trivial. Most of these studies estimate the coupling between function and structure based on the correlation coefficient across all connections. This approach ignores the network organisation of the human brain that results in multivariate interactions among connections, ie. the influence that one connection exerts over another [14, 49]. Our work can account for these because we have built a multivariate intersubject prediction framework that could capture the influences one connection has over another.

Interpreting structural connectivity correctly is challenging for several reasons and below we discuss further some of these challenges with relation to our work. Firstly, more than 90% of white matter voxels contain information from multiple distinct fibre populations [50]. Therefore, constructing structural connectivity maps by averaging DTI-derived measures, such as FA and MD, across streamlines is not accurate. A number of alternative approaches have been proposed, such as the apparent fibre density, which is based on the assumption that intra-axonal water is restricted in the radial direction [50]. Furthermore, the hindrance-modulated orientation anisotropy has been introduced, which is defined as the absolute amplitude of each lobe of the fiber orientation distribution [51]. There is a fundamental difference between these previous approaches and our work. Both of these measurements are derived from the high b-value HARDI shell alone, and share much of the information contained already within the derived tractogram. Here we aimed towards a more independent measure of tissue microstructure based on NODDI, which is a tissue compartment-based modeling technique that makes use of multi-shell diffusion data.

On the other hand, it has been suggested to retrospectively improving the reconstruction of streamlines to address the limited biological accuracy of structural brain networks derived from DWI [52]. Selective filtering of the streamlines derived from the tractogram is applied, so that the fit between the streamline reconstruction and underlying diffusion images is improved. This approach has some similarities with the idea behind global tractography [53], which fits the signal across the whole streamline and results in more accurately structural brain networks at the expense of complexity and time required to run the algorithm [54]. This limits the number of streamlines that can be generated. Therefore, it has been suggested to generate a large number of streamlines with the traditional approaches and then choose a subset that best matches the diffusion signal, to improve the accuracy of the derived structural connectomes. Similarly, to apparent fiber density and the hindrance-modulated orientation anisotropy, the derived tractograms does not result in independent measurements of structural brain connectivity. Furthermore, this approach cannot improve the false negative rate, which results in sparse inter-hemispheric connectivity.

Independence between the tractograms and the microstructural measurements becomes important when we examine their pairwise relationship, Fig 7. These correlations should be indicative of the inherent relationship of the microstructural indices. Subsequently, the identification of relevant connections derived for each microstructural index, Figs 5 and 6, reflects how well the index can characterize the underlying connection independently of the tractogram used. We speculate that some indices may be able to characterize some functional connections better than others and vice-versa. Nevertheless, we do not claim that our results are fully independent of the tractograms. Future work should focus on eliminating false negative connections and investigating how this affects the results. Furthermore, our work does not aim to exhaustively compare microstructural indices and it likely that other metrics could provide more direct measurements [50, 51]. Here, we have set a general modeling framework of functional from structural connectivity that could facilitate towards this direction.

An important limitation in our work is that we have excluded the connections that were absent in one or more subjects, as it is unclear how to handle missing values in the sCCA framework. This mostly affects interhemispheric connectivity, because interhemispheric connections are long connections. This is in agreement with literature that reports that tractography algorithms underestimate long-range connectivity [42, 55, 56]. These studies demonstrated that probabilistic tractography algorithms favour the shortest, simplest and straightest paths. We should point out that this lack of inter-hemispheric structural connectivity is not unique to the tractography algorithm we have used in this study. In fact, it has been also replicated independently with other state-of-the-art tractography approaches that model the fibre orientation density function from diffusion-weighted MRI data using constrained spherical deconvolution (CSD) [57].

We used the two compartments ball-and-sticks model. This fiber orientation model is still considered a state-of-the-art approach and it compares well with CSD approaches [58]. Although, spherical deconvolution approaches have higher fiber detection rate, we do not expect substantial changes to the results. Moreover, our previous work has shown that there is considerable agreement between the connectomes derived with CSD approaches and the ball-and-sticks connectomes [59].

Partial volume effects caused by non-WM tissue at the boundaries between WM and GM are apparent in both CSD and ball-and-sticks approaches [60]. Recently, multi-shell CSD methods have been proposed to address limitations of CSD in voxels containing GM and cerebrospinal fluid [61]. This is likely to affect the termination of streamlines. In our study, we used prior anatomical knowledge based on T1-weighted images, which have been preprocessed with FreeSurfer to accurately segment white matter, gray matter and CSF, and parcellate gray matter regions of interest. Subsequently, we transfer this parcellation to Diffusion Weighted space with non-rigid registration. Streamlines are terminated when they reach a gray matter region based on anatomical prior similar to previous work. This is a relatively accurate approach that has been used in several clinical studies. It has been reported that further upsampling in the diffusion weighted images reduces partial volume effects [62]. Their results show to improve the recovery of streamlines unilaterally in specific areas. Nevertheless, this funding could not explain why inter-hemispheric connectivity is sparser than intra-hemispheric connectivity.

We chose to use only cortical gray matter regions for three major reasons. Firstly, adding more brain regions to the brain connectivity model increases the complexity quadratically, whereas the number of available observations/subjects remains the same. Therefore, including more regions it is likely to result in substantial reduction of the sensitivity of the proposed framework. Secondly, the accuracy of source reconstruction of EEG signals in deep gray matter regions is substantially less accurate, with a major decrease to the signal to noise ratio [17]. This will further complicate the interpretation of the results. Finally, appropriate determination of streamline termination in deep gray matter region is also more challenging. Therefore, limiting the current study to cortical regions is a reasonable choice.

Although, the signal to noise ratio (SNR) at 1.5T is less than in higher fields, it is unlikely to cause a large difference in the results. Firstly, the SNR in fMRI is dominated by factors associated with temporal stability and physiological noise, which reduces the advantage of higher field strengths. In fact, fMRI signal degradation due to the EEG cap is lessened at 1.5T [63]. The low magnetic field at 1.5T is also advantageous for EEG quality due to the reduction in cardiac-related and motion artefacts. Note that our specialized EEG equipment is designed to limits artefacts at 1.5 and 3T to the skull and skin [64, 65]. Finally, one of the advantages of 1.5T for diffusion imaging is the longer *T*_2_ than at 3 T. This turns out to not only compensate for the reduction in SNR due to field strength difference, but also supports the larger b-value of 2400, higher than the corresponding value at 3 T, which is 2000.

## Supporting Information

S1 Data The supplementary data file contains. a) Functional connectivity matrices, both fMRI and EEG connectomes across all subjects, in the form of precision matrices and b) structural brain connectivity matrices across all subjects and microstructural indices. Columns and rows are named according to the corresponding brain region that represent.
(ZIP)
