# Supplementary material for: NODDI and Tensor-Based Microstructural Indices as Predictors of Functional Connectivity
Source: PLoS One. 2016 Apr 14;11(4):e0153404. doi: 10.1371/journal.pone.0153404 (PMC4831788; doi:10.1371/journal.pone.0153404)
Supplement: S1 Data — a) Functional connectivity matrices, both fMRI and EEG connectomes across all subjects, in the form of precision matrices and b) structural brain connectivity matrices across all subjects and microstructural indices. Columns and rows are named according to the corresponding brain region that represent. (ZIP) [file pone.0153404.s001.zip › DATA/readme.rtf]

This folders contain the datasets associated with PLOS ONE submission ‘NODDI and tensor-based microstructural indices as predictors of functional connectivity’.
Both EEG and fMRI connectomes are represented with the precision matrix of each subject.
Abbreviations correspond to the same abbreviations adopted in the paper. 
Columns and rows are named according to the corresponding brain region that represent. The correspondence between number codes and brain regions is represented in the lookup table ‘parcellation.lut’, which has been adapted from Freesurfer Desikan-Killiany atlas.
